# Machine learning prognosis model based on patient-reported outcomes for chronic heart failure patients after discharge

**DOI:** 10.1186/s12955-023-02109-x

**Published:** 2023-03-29

**Authors:** Jing Tian, Jingjing Yan, Gangfei Han, Yutao Du, Xiaojuan Hu, Zixuan He, Qinghua Han, Yanbo Zhang

**Affiliations:** 1 Department of Cardiology, the 1st Hospital of Shanxi Medical University, 85 South Jiefang Road, Taiyuan, Shanxi Province 030001 China; 2 Shanxi Provincial Key Laboratory of Major Diseases Risk Assessment, 56 South XinJian Road, Taiyuan, Shanxi Province 030001 China; 3 Department of Health Statistics, School of Public Health, Shanxi Medical University, 56 South XinJian Road, Taiyuan, Shanxi Province 030001 China; 4 Shanxi University of Chinese Medicine, 121 University Street, Jinzhong, Shanxi Province 030619 China

**Keywords:** Patient-reported outcome, Chronic heart failure, Prognosis model, Machine learning

## Abstract

**Background:**

Patient-reported outcomes (PROs) can be obtained outside hospitals and are of great significance for evaluation of patients with chronic heart failure (CHF). The aim of this study was to establish a prediction model using PROs for out-of-hospital patients.

**Methods:**

CHF-PRO were collected in 941 patients with CHF from a prospective cohort. Primary endpoints were all-cause mortality, HF hospitalization, and major adverse cardiovascular events (MACEs). To establish prognosis models during the two years follow-up, six machine learning methods were used, including logistic regression, random forest classifier, extreme gradient boosting (XGBoost), light gradient boosting machine, naive bayes, and multilayer perceptron. Models were established in four steps, namely, using general information as predictors, using four domains of CHF-PRO, using both of them and adjusting the parameters. The discrimination and calibration were then estimated. Further analyze were performed for the best model. The top prediction variables were further assessed. The Shapley additive explanations (SHAP) method was used to explain black boxes of the models. Moreover, a self-made web-based risk calculator was established to facilitate the clinical application.

**Results:**

CHF-PRO showed strong prediction value and improved the performance of the models. Among the approaches, XGBoost of the parameter adjustment model had the highest prediction performance with an area under the curve of 0.754 (95% CI: 0.737 to 0.761) for death, 0.718 (95% CI: 0.717 to 0.721) for HF rehospitalization and 0.670 (95% CI: 0.595 to 0.710) for MACEs. The four domains of CHF-PRO, especially the physical domain, showed the most significant impact on the prediction of outcomes.

**Conclusion:**

CHF-PRO showed strong prediction value in the models. The XGBoost models using variables based on CHF-PRO and the patient’s general information provide prognostic assessment for patients with CHF. The self-made web-based risk calculator can be conveniently used to predict the prognosis for patients after discharge.

**Clinical Trial Registration:**

URL: http://www.chictr.org.cn/index.aspx; Unique identifier: ChiCTR2100043337.

**Graphical abstract:**

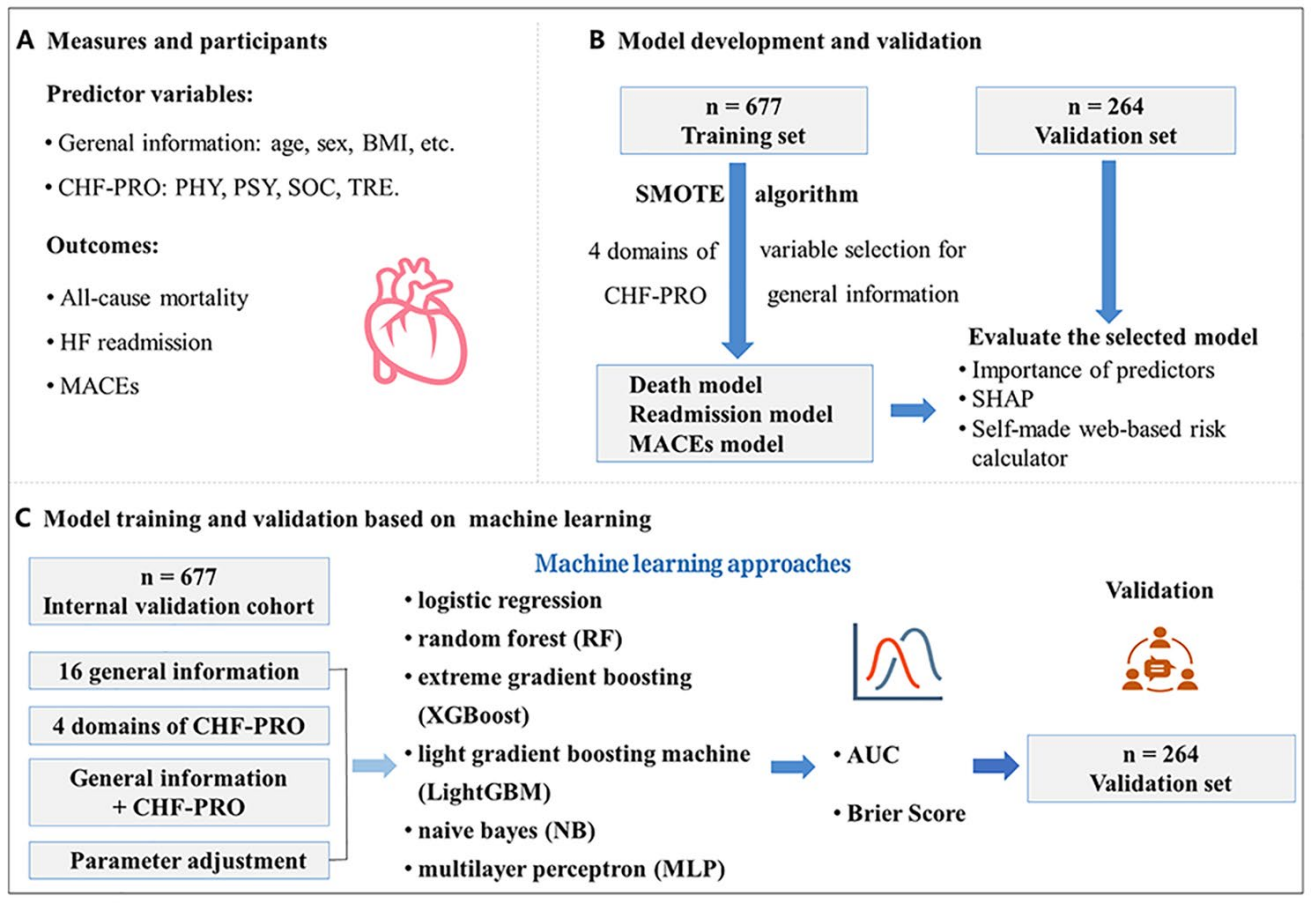

**Supplementary Information:**

The online version contains supplementary material available at 10.1186/s12955-023-02109-x.

## Introduction

Chronic heart failure (CHF) is the terminal stage of cardiovascular diseases. The high mortality and readmission rates put a heavy burden to families and societies [1, 2]. Accurate prediction of the prognosis of patients with CHF can assist physicians in making treatment decisions and improve the prognosis of the patients. Most prediction models currently depend on clinical indicators and biomarkers obtained during the hospitalization [3, 4]. However, it is impossible for patients and their families to obtain parts of the necessary clinical indicators after discharge. Moreover, the prediction models still depend on unchanged baseline data even after the patients are out-of-hospital, which reduces the predictive performance of the models. Therefore, models based on the data that can be obtained outside hospitals and reflects the changes of disease and patients’ status will be more conducive to assess the prognosis of CHF patients after discharge and guide chronic disease management.

Patient-reported outcomes (PROs) are presented in the form of self-filling scales and could be obtained conveniently for patients after discharge [5]. The guidance of the US Food and Drug Administration claimed that PROs should be used as one of the most important endpoints for evaluating the clinical trials of new drugs [5]. The importance of PROs has gradually been realized by physicians and researchers. Considering CHF, PROs have a higher possibility of recording the effects of the course of the illness than other chronic diseases [6]. Moreover, studies showed that PRO was closely associated with the prognosis of CHF [7–10]. The 2022 American Heart Association guideline for management of HF recommended that standardized assessment of patient-reported outcomes was able to provide incremental information for patients’ prognosis [7]. Therefore, for patients lacking clinical indicators or biomarkers after discharge, we could use PROs as alternative prognosis indicators.

PROs covered the domains of physiological symptoms, psychology, social support, treatment compliance, and satisfaction [5]. Any alteration in the patient’s condition may be expected to be noted in PROs, which makes the data of PROs complex and uncertain. Machine learning (ML), such as random forest and extreme gradient boosting (XGBoost), is currently considered to be data analysis methods with high predictive performance in clinical predictive models. Therefore, in this study for CHF patients who lacked some clinical indicator after discharge, we tried to applied a PRO as an alternative to establish a prognosis model via machine learning approaches. The model will predict the risk of death and HF readmission for those patients and facilitate appropriate individual patient management.

## Methods

### Setting and participants

**Fig. 1:**
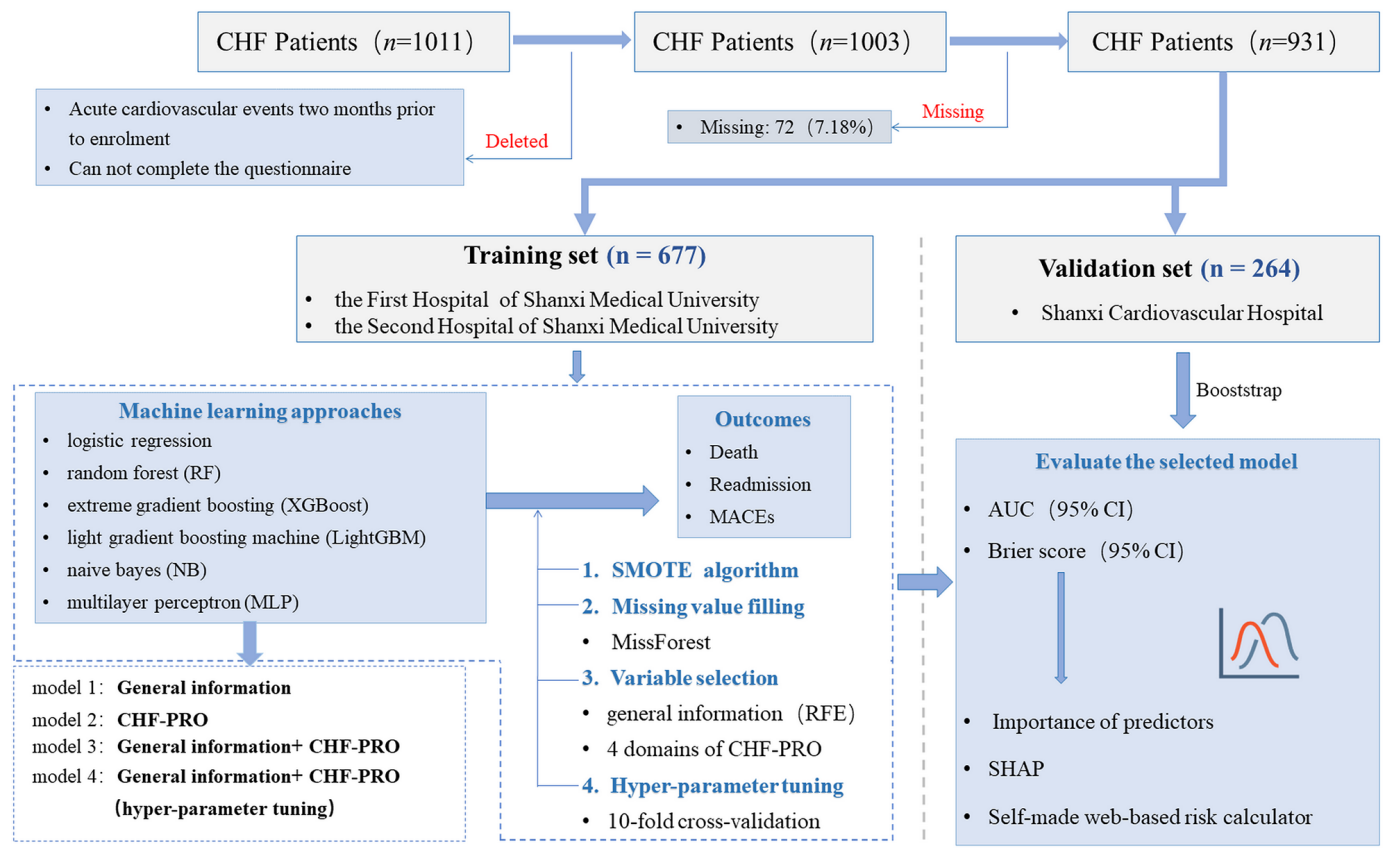
Flowchart of the Study

This study was designed as a multi-center, prospective cohort study. The checklist of items for TRIPOD statement of this study was shown in Table S1. Patients from three medical centers in Shanxi Province of People’s Republic of China were enrolled between July 1, 2017 and June 30, 2019. A total of 1011 patients hospitalized for CHF were enrolled in the study. Among them, 1003 (99.21%) patients completed the CHF-PROM, and 941 (93.08%) of them attended the follow-up examinations. The flow diagram is shown in Fig. 1.

Eligibility requirements included only patients who were diagnosed with HF according to the ESC guideline [1] and classified as functional class II-IV according to the New York Heart Association (NYHA). Patients who had suffered acute cardiovascular events two months prior to enrolment or were not able to complete the questionnaire owing to intellectual disabilities were excluded. All subjects provided informed consent for inclusion before they participated in the study. The study was conducted in accordance with the Declaration of Helsinki, and the protocol was approved by Institutional Review Board of Shanxi Medical University.

### Measures

General information and PROs of patients were collected during hospitalization. All participants who reported PROs were followed-up after discharge at 1, 3, and every 6 months thereafter by telephone to obtain the information on outcomes. To guarantee the quality of the data collected, all questionnaires were collected by professionally trained individuals.

#### General information

The following demographic and clinical information were collected as general information in our study: age, sex, body mass index (BMI), occupation, level of education, health insurance, history of smoking and alcohol drinking, family history, blood pressure, heart rate, NYHA class, and severe comorbidities.

The following points were considered when collecting the general information:

- Health insurance was classified as either urban or rural health insurance in our study based on the national policies of the People’s Republic of China. Urban health insurance covers about 80% of hospitalization expenses, whereas rural health insurance covers only 60%.
- Comorbidities included coronary heart disease, valvular heart disease, hypertension, diabetes mellitus, atrial fibrillation, chronic obstructive pulmonary disease, and renal insufficiency [1].

#### CHF-PRO

The patient-reported outcome of chronic heart failure measure (CHF-PROM) developed by the authors’ research group was used in this study [11]. The structure of CHF-PROM is presented in Table S2. This questionnaire contains 57 items and covers four domains of patients’ health status including the physical domain (PHY), psychological domain (PSY), social domain (SOC), and therapeutic domain (TRE).

#### Outcomes

The outcomes of interest included all-cause mortality, HF hospitalization, and major adverse cardiovascular events (MACEs) throughout the two-year follow-up. All-cause mortality was defined as death due to any cause. HF hospitalization was defined as an admission of more than 24 h with exacerbation of HF after the index admission. HF as the cause for hospitalization was judged by professionals during follow-up and confirmed by ICD-10 diagnosis of HF as the patient’s primary diagnosis. MACEs in the study comprised all-cause mortality and HF hospitalization as mentioned above.

### Feature selection and data preprocessing

Training set was performed via a cohort of 677 patients of the First Hospital and the Second Hospital of Shanxi Medical University, and validation set was completed via a cohort of 264 patients of Shanxi Cardiovascular Hospital. Missing value filling was performed in the training set and the validation set, respectively. In the training set, the parameter adjustment was performed with 10-fold cross-validation, while in the validation set, external validation and 95% confidence interval estimation were conducted via Bootstrap.

The independent variables of our study comprised 24 general information data points and the 4 domains of CHF-PRO (PHY, PSY, SOC, and TRE). All of the continuous variables were presented as means ± standard deviations (SD) or median ± interquartile range. The categorical variables were expressed as *n* (%). Continuous variables were compared using independent *t*-tests for normality distribution and rank-sum test for non-normality distribution. The chi-square test was used to compare the rates. All tests were two-sided, and *P* < 0.05 was considered as statistical significance.

The variables that missing more than 30% were deleted [12, 13]. For the data missing less than 30%, we added it with missForest [14] which was completed by R version 4.0.5 (Lucent Technologies, Murray Hill, NJ, USA). In addition, Cronbach’s *α* coefficient was used to assess the data quality of the CHF-PRO. Since CHF-PRO comprised the complete entity, we used all the 4 domains of it as the prediction features, and recursive feature elimination (RFE) method was used to select the variables of general information. The gain information was used to implement the process of feature-ranking.

### Processing of imbalanced data

In this study the ratio between mortality rate and survival rate was rougthly 1:13, which was a severe unbalance distribution of samples. Accordingly, were the ratios of readmission (1:3) and MACEs (1:2) were observed. Therefore, we applied synthetic minority over-sampling technique algorithm (SMOTE) to resolve the imbalanced distributions of the outcomes [15].

### Machine learning model approaches

Six ML approaches were used to train prediction models for mortality and HF hospitalization over the two years of follow-up. 10-fold cross-validation was used to select the value of the training parameters in an attempt to minimize the model deviance. The approaches applied in the study included logistic regression, random forest (RF) classifier, XGBoost, light gradient boosting machine (LightGBM), naive bayes (NB), and multilayer perceptron (MLP). Logistic regression (LR) was performed in this study as the basic model for the prediction. RF is a supervised ensemble learning method and based on decision trees that were built from the variable set. RF performs well in solving the overfitting problem of unbalanced data [16]. XGBoost is another ensemble tree algorithm. It is composed of a series of base classifiers which are linearly superimposed to optimize the algorithm after they are determined [17]. The LightGBM model is a type of optimized gradient boosting decision tree and can reduce the calculation amount of the structure fraction [18]. NB is based on Bayesian decision theory and Bayesian networks, and it is known to be insensitive to missing data [19], exhibit stable classification efficiency, and can process multiple classification tasks. Therefore, we were able to obtain better classification results by using NB [20]. MLP is a commonly used feedforward artificial neural network. It can adjust the weight of connections between neurons to obtain an output value which is equal to or close to the target value [21].

All models were constructed in four phases. First, they were constructed using only general information as predictors (model 1). Second, they were modified using four domains of CHF-PRO (model 2). Third, four domains of CHF-PRO were added to the general information predictors (model 3). Finally, we performed the parameter adjustment for model 3 (model 4). In the fourth step, we adjusted the parameters through learning curve and grid search to obtain the optimal configuration for each ML algorithm. Various software packages in the Python 3.7 that were used to perform the analysis and the optimized hyperparameters of each of the ML algorithms were shown in Table S3. We traversed all the combinations of parameters for each ML algorithm, and then determined the prediction results using 10-fold cross validation. The area under curve (AUC) was used to assess the model fitness function of variables.

### Evaluation of candidate machine learning models

Prediction performance of all model approaches was evaluated using the following parameters for the validation data from Shanxi Cardiovascular Hospital:

- AUC was used to evaluate the discrimination ability of the predictive models. A 95% confidence interval (CI) of AUC was calculated in this study.
- Brier score was used to assess the accuracy of the probability of the models and is defined as the mean squared differences between actual binary outcomes and predicted probabilities [22]. It ranges from 0 to 1.00, with a score of 0 indicating perfect prediction.
- Calibration curves were used to determine the proximity between the predicted probabilities.

and observed probabilities. of outcomes for the optimal models.

The model with the optimal parameters was selected as the final model to the corresponding outcome for further analysis.

### Evaluation of the selected machine learning models

#### Feature importance

The importance of each variable was ranked in the best performing models for death, rehospitalization, and MACEs. We applied a map of feature importance to represent the result.

#### Model interpretation

The SHAP method is a novel approach to explain various black boxes of ML models and has been validated in its interpretability performance [23]. Therefore, we applied SHAP to provide the interpretation for our prediction models with the contributing risk factors that lead to death and rehospitalization in patients with CHF. Shap packages in the Python 3.7 was used for this analysis.

To facilitate the application of the prediction model, Python 3.7 software was used to establish the self-made web-based risk calculator for patients with CHF. We transformed the models with the best verification to the self-made web-based risk calculator.

## Results

### Characteristics and candidate variables

During the follow-up period, 65 (6.91%) patients died, and 268 (28.48%) patients were re-hospitalized due to exacerbated HF. Table S4 – S7 summarizes the baseline characteristics of the patients. Cronbach’s α coefficients for the PHY, PSY, SOC, and TRE scores, and the overall scale were 0.901, 0.929, 0.850, 0.856, and 0.914, respectively.

### Comparison of modeling approaches

The results of the models constructed by four steps and six ML algorithms are shown in Table 1. Taking the XGBoost model results as an example, the model 2 based on four domains of CHF-PRO showed better discrimination than the model 1 which used 16 indicators of general information (AUC: 0.601 (0.598, 0.604) vs. 0.519 (0.518,0.522), *P* < 0.001). The model 3 based on four domains of CHF-PRO and general information showed better discrimination than the model 1 which only used general information (AUC: 0.607(0.595,0.608) vs. 0.519(0.518,0.522), *P* < 0.001). Adjustment of parameters (Model 4) further improved the performance of the model 3.

**Table 1:** Comparison of Models in Predicting Outcomes in Patients With Heart Failure

	General information (Model 1)			CHF-PRO (Model 2)			General information + CHF-PRO (Model 3)			Model 3 + Parameter adjustment (Model 4)		
	BS	AUC	*P*	BS	AUC	*P*	BS	AUC	*P*	BS	AUC	*P*
All-Cause death												
XGBoost	0.205	0.519 (0.518,0.522)	Reference	0.237	0.601 (0.598, 0.604)	Reference	0.080	0.607 (0.595,0.608)	Reference	0.174	0.754 (0.737,0.761)	Reference
LightGBM	0.110	0.520 (0.518,0.525)	< 0.001	0.205	0.621 (0.611,0.637)	< 0.001	0.076	0.613 (0.594,0.618)	< 0.001	0.095	0.733 (0.713,0.754)	< 0.001
RF	0.106	0.547 (0.566,0.611)	< 0.001	0.152	0.590 (0.595,0.598)	< 0.001	0.091	0.608 (0.599,0.621)	< 0.001	0.371	0.709 (0.690,0.719)	< 0.001
Logistic	0.083	0.681 (0.673,0.683)	< 0.001	0.091	0.689 (0.683,0.696)	< 0.001	0.095	0.720 (0.710,0.734)	< 0.001	0.087	0.742 (0.727,0.745)	< 0.001
NB	0.080	0.500 (0.500,0.500)	< 0.001	0.894	0.514 (0.514,0.514)	< 0.001	0.894	0.514 (0.514,0.514)	< 0.001	0.356	0.658 (0.657,0.666)	< 0.001
MLP	0.364	0.509 (0.504,0.514)	< 0.001	0.392	0.678 (0.670,0.692)	< 0.001	0.413	0.645 (0.593,0.662)	< 0.001	0.288	0.746 (0.741,0.752)	< 0.001
**HF readmission**												
XGBoost	0.322	0.590 (0.588,0.594)	Reference	0.415	0.535 (0.532,0.538)	Reference	0.277	0.644 (0.641,0.646)	Reference	0.235	0.718 (0.717,0.721)	Reference
LightGBM	0.288	0.548 (0.544,0.551)	< 0.001	0.405	0.519 (0.512,0.525)	< 0.001	0.254	0.610 (0.608,0.615)	< 0.001	0.231	0.704 (0.654,0.733)	< 0.001
RF	0.269	0.552 (0.540,0.556)	< 0.001	0.349	0.539 (0.541,0.544)	< 0.001	0.265	0.580 (0.564,0.604)	< 0.001	0.216	0.707 (0.702,0.710)	< 0.001
Logistic	0.458	0.639 (0.636,0.642)	< 0.001	0.278	0.585 (0.580,0.586)	< 0.001	0.405	0.665 (0.661,0.667)	< 0.001	0.307	0.693 (0.686,0.701)	< 0.001
NB	0.261	0.500 (0.500,0.500)	< 0.001	0.265	0.508 (0.509,0.512)	< 0.001	0.261	0.498 (0.498,0.498)	< 0.001	0.390	0.673 (0.665,0.685)	< 0.001
MLP	0.409	0.585 (0.580,0.594)	< 0.001	0.485	0.562 (0.551,0.579)	< 0.001	0.390	0.615 (0.612,0.619)	< 0.001	0.220	0.690 (0.640,0.712)	< 0.001
**MACEs**												
XGBoost	0.405	0.527 (0.523,0.531)	Reference	0.439	0.540 (0.538,0.544)	Reference	0.318	0.600 (0.588,0.597)	Reference	0.364	0.670 (0.595,0.710)	Reference
LightGBM	0.443	0.515 (0.512,0.521)	< 0.001	0.420	0.519 (0.508,0.530)	< 0.001	0.367	0.580 (0.579,0.607)	< 0.001	0.348	0.620 (0.594,0.644)	< 0.001
RF	0.383	0.527 (0.525,0.530)	> 0.999	0.410	0.540 (0.545,0.546)	> 0.999	0.383	0.552 (0.540,0.557)	< 0.001	0.356	0.666 (0.641,0.680)	0.003
Logistic	0.470	0.593 (0.580,0.611)	< 0.001	0.358	0.607 (0.605,0.609)	< 0.001	0.424	0.613 (0.597,0.622)	< 0.001	0.402	0.629 (0.612,0.645)	< 0.001
NB	0.337	0.500 (0.500,0.500)	< 0.001	0.337	0.497 (0.499,0.500)	< 0.001	0.337	0.497 (0.497,0.497)	< 0.001	0.455	0.657 (0.646,0.669)	< 0.001
MLP	0.576	0.530 (0.519,0.547)	< 0.001	0.394	0.619 (0.605,0.624)	< 0.001	0.394	0.575 (0.570,0.578)	< 0.001	0.284	0.670 (0.647,0.692)	> 0.999

AUC, area under curve; BS, brier score; CHF-PRO, chronic heart failure - patient reported outcome; LightGBM, light gradient boosting machine; MACEs, major adverse cardiovascular events; MLP, multilayer perceptron; NB, naive bayes; RF, random forest; XGBoost, extreme gradient boosting

**Fig. 2:**
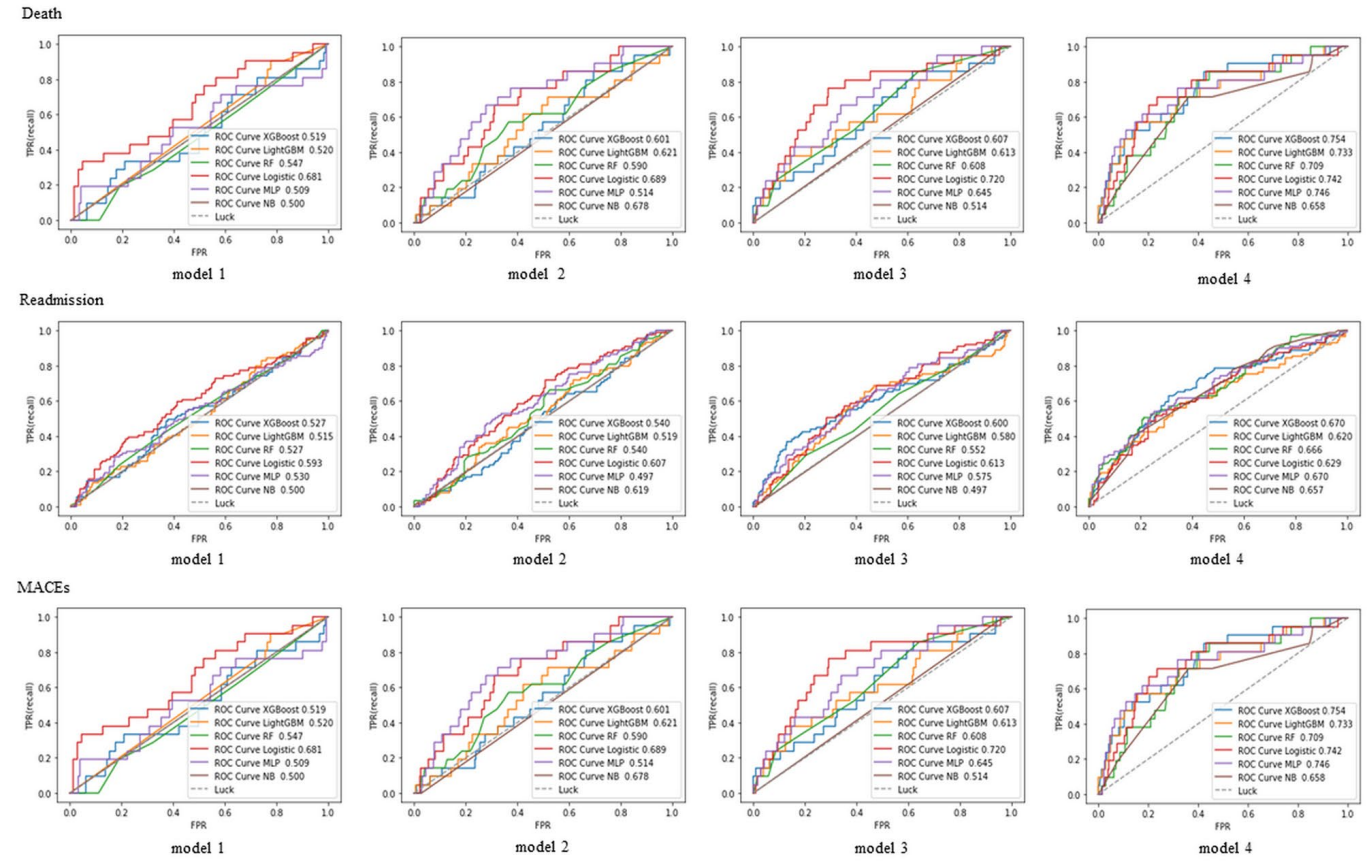
ROC curve of 3 outcomes. The ROC results of six machine learning models for death, readmission and MACEs. For each outcome, models built by general information are shown in model 1, by four domains of CHF-PRO are shown in model 2, by general information and CHF-PRO are shown in model 3, and by general information and CHF-PRO and adjusted parameters are shown in model 4. FPR, false positive rate; TPR, true positive rate

Among the six ML approaches, XGBoost had the highest predictive performances for all three outcomes, which was closely followed by RF. The XGBoost models achieved a mean AUC of 0.754 (95% CI: 0.737 to 0.761) for death, 0.718 (95% CI: 0.717 to 0.721) for HF rehospitalization and 0.670 (95% CI: 0.595 to 0.710) for MACEs. Compared with the Logistic model, XGBoost showed significant improvement (AUC: 0.754 (0.737,0.761) vs. 0.742 (0.727,0.745), *P* < 0.001). In contrast, the NB model exhibited an AUC of 0.658 (95% CI: 0.657 to 0.666) and 0.673 (95% CI: 0.665 to 0.685) for death and HF rehospitalization, respectively. The ROC comparison among the models is shown in Fig. 2. The Brier scores of XGBoost showed a moderate effect among these models, with 0.174 for death, 0.235 for readmission, and 0.364 for MACEs as shown in Table 1. The calibration curves of XGBoost models performed well for death and readmission, but is unsuitable for MACEs, as presented in Figure S1.

### Evaluation of XGBoost models

#### Importance of predictors

**Fig. 3:**
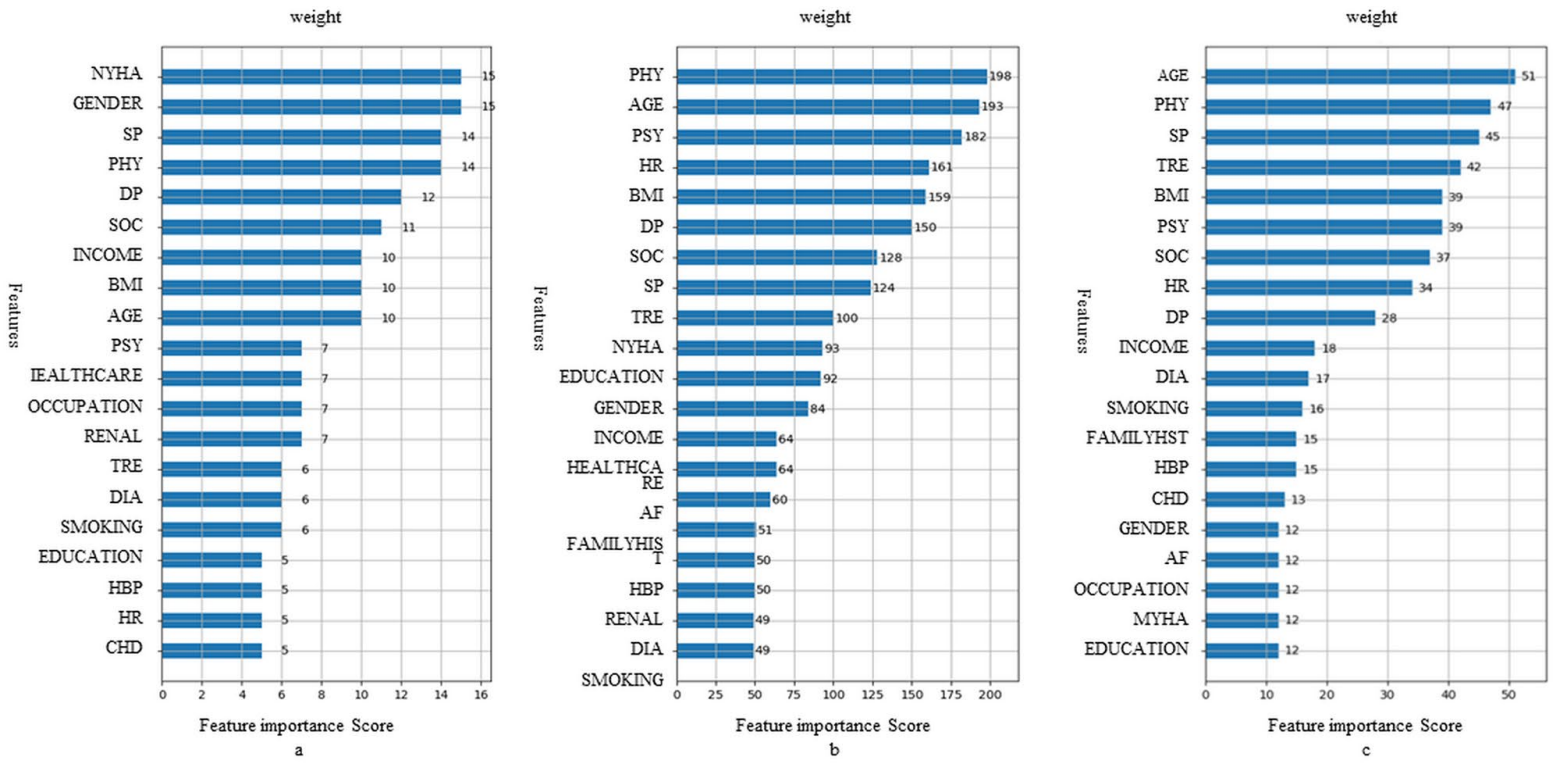
The weight of predictors in XGBoost models of general information and CHF-PRO. The weight of predictors in XGBoost models of (a) all-cause death, (b) HF readmission and (c) MACEs. The number at the end of the horizontal axis of each variable indicates its weight in the model. AF, atrial fibrillation; BMI, body mass index; CHD, coronary heart disease; DIA, diabetes; DP, diastolic pressure; FAMILYHISY, family history; HBP, hypertension; HR, heart rate; Renal, renal insufficiency; SP, systolic pressure

The importance of the variables was ranked in descending order for each outcome of the XGBoost models. Figure 3 contains the importance of predictors measured by XGBoost based on general information and CHF-PRO. Among these predictors, the domains of CHF-PRO, especially the physical domain, played more important roles than most of the general information.

#### Model interpretation

To highlight the clinical utility and translational impact of such predictions in chronic care management, we presented cases of patients with the different end-points.

**Fig. 4:**
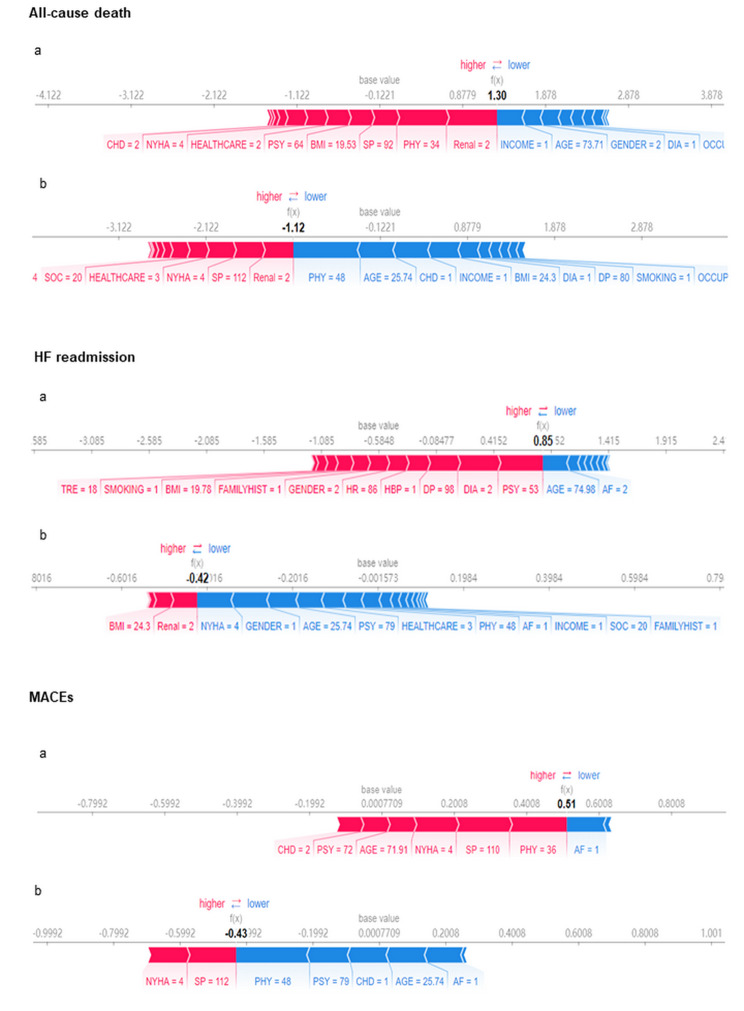
Explanation of the prediction results for specific instances. This figure shows the explanation for patients with the corresponding events (a) and patients without the corresponding events (b). The base values are the average values of predictive models; and the f(x)s are the predicted risks. The bars in red and blue represent the risk factors and protective factors, respectively; the longer bars represent greater feature importance. AF, atrial fibrillation; BMI, body mass index; CHD, coronary heart disease; DIA, diabetes; DP, diastolic pressure; HBP, hypertension; FAMILYHISY, family history; HR, heart rate; Renal, renal insufficiency; SP, systolic pressure

For the cases shown in Fig. 4, the SHAP values of CHF patients with death, re-hospitalization, and MACEs were higher than those of patients without end-point events. Take Fig. 4A as an example, the model was used to assess the death risk of a 74-year-old woman with coronary heart disease, diabetes and chronic renal insufficiency. The general conditions included low-income, a manual worker, and the rural medical insurance. For information of CHF-PRO, the score of PHY and PSY were 34 and 64, respectively. The total SHAP value *f* (x) of the patient was 1.30. The positive effect (red) was greater than the negative effect (blue), indicated the high risk of death.

#### Clinical application of the model

A self-made web-based risk calculator was established to facilitate the clinical application. The left side of the calculator is the input window. In the window, the continuous variables of patients were assigned by dragging, and categorical variables were dropped-down to select. The right side is the result output window. According to the results, the two-year risk rates for mortality, rehospitalization and the MACEs were 20.16%, 55.22%, and 94.81%.

Insert Fig. 5 here.

**Fig. 5:**
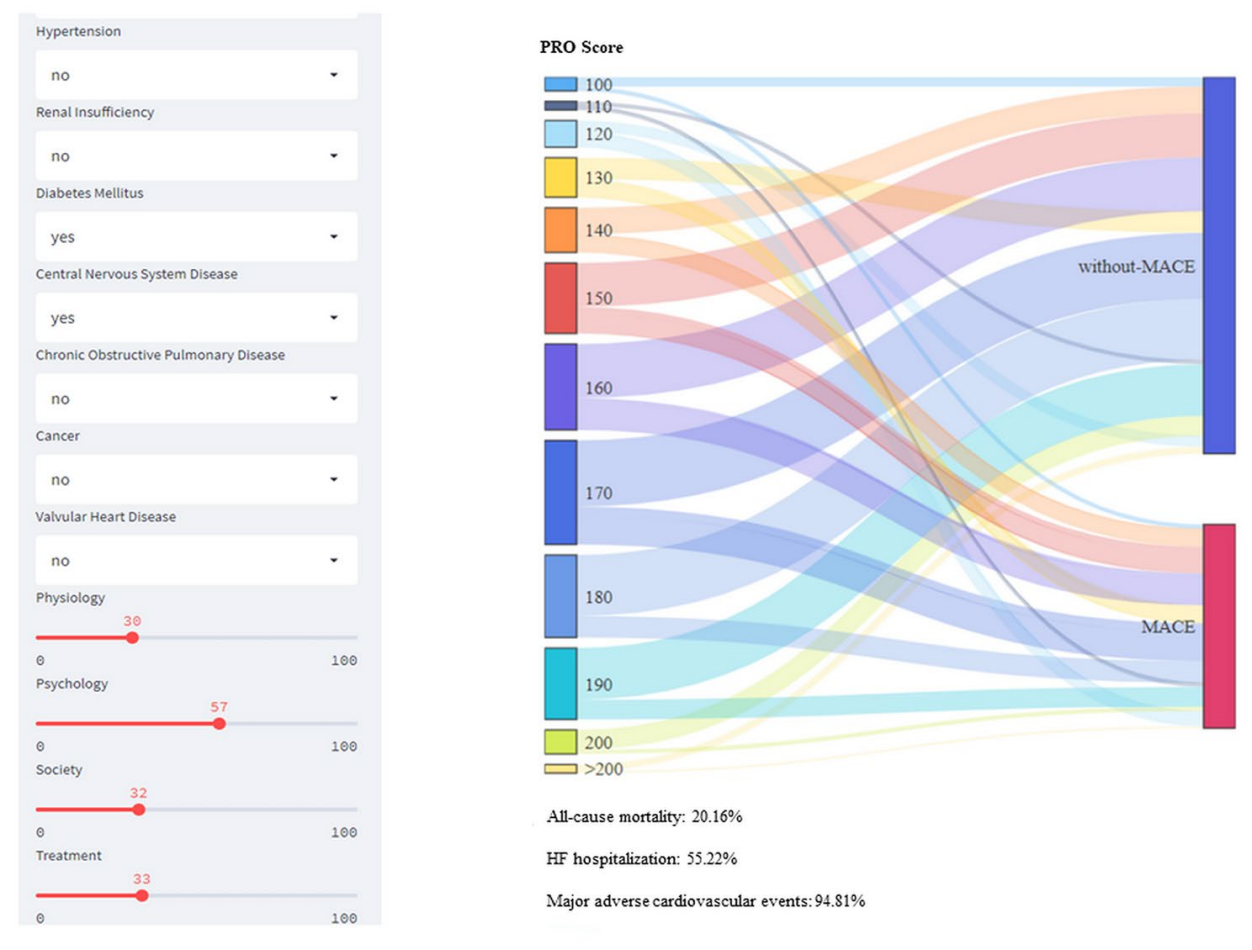
Self-made web-based risk calculator for specific instances. On the left side of the system is the variable input module, where continuous variables can be assigned by dragging, and categorical variables such as gender can be drop-down to select variable assignment. On the right is the results output window. Based on the results, the two-year mortality rate, re-hospitalization rate and MACEs incidence rate of patients that met the input conditions were 7.81%, 49.23% and 64.8% respectively

## Discussion

Evaluation of the prognosis of patients with CHF is critical to allow clinicians to select appropriate treatment strategies accurately. In this study, the PRO-driven models that we developed and validated showed good performance for event prediction in patients with CHF. Importantly, these models only require variables can be implemented after discharge. Moreover, we introduced SHAP approach and established a self-made web-based risk calculator, which could predict the prognosis of each individual, to explain the black box of ML models. To our knowledge, this is one of only a few studies that focus on prognosis models in CHF mainly using information gathered through PROs.

This study demonstrated that CHF-PRO had high predictive value for mortality and HF readmission in patients with CHF. Previous studies have also confirmed that PRO is an essential prognosis indicator for HF even adjusting for traditional variables [6, 8]. Moreover, PRO has also been applied as one of the predictor variables to establish the prognosis model of CHF. Different from the previous studies, we constructed prognosis models primarily based on the information of CHF-PRO and obtained a good predictive effect in this study. This is consistent with our previous study concerning a readmission model through logistic regression [24]. The data on all the indicators applied in this study could be obtained through telephone or self-test, which is expected to provide a feasible prediction and guidance tool for the out-of-hospital management of patients with CHF. Among the four domains of CHF-PRO, physical status was the strongest predictor in this study. In addition, the remaining subscales of CHF-PRO also proved to be important for accurate prediction. This supports the findings of previous studies [25]. Providing relief for the physical symptoms is one of the most important goals of CHF treatment, but the psychological status and social factors of patients with CHF should also be considered during the clinical application.

We found that ML methods failed to improve the discrimination ability of logistic regression. A meta-analysis that used AUC to measure the performance of models from 71 studies confirmed that there was no evidence of superior performance of ML over logistic regression [26]. However, in this study we found that the parameter adjustment significantly improved the accuracy of probability and discrimination of ML, except that in logistic regression. This observation may be attributed to the logistic regression being specialized in linear data processing, and the possible adjustments to parameters are limited. The result reminds that when applying the ML methods to the complex data, we could improve the model performance through parameter adjustment. Among all the ML, the XGBoost algorithm had the highest predictive performance in our study. XGBoost is a decision-tree-based algorithm and composed of a series of base classifiers such as decision tree, k-nearest neighbor, support vector machines, and logistic regression. The base classifiers are linearly superimposed to optimize the algorithm after they are determined [17]. Studies showed the XGBoost model offers strong generalization ability, high scalability, and fast computing speed in model building [27]. XGBoost typically shows outstanding performance when dealing with complex problems. It is suitable for almost all types of complex classification problems [28–30] and showed good predictive value in many studies on prognosis models [27, 31].

Additionally, the black box of ML was opened by interpretability techniques in this study. Through SHAP algorithm, we can understand the relationship between predictors and outcomes in the XGBoost models. The contributions of the variables for each individual could be obtained from the result of SHAP, which helps better understand the decision-making process of the model and facilitate its use in clinical setting [32]. Meanwhile, a self-made web-based risk calculator was established in this study. Through the calculator, we could easily get the incidence rates of outcomes and identify patients with the high risk. From these two interpretable algorithms, we can identify both high-risk factors and high-risk individuals, which provided unique tools to better guide clinical decision making.

Despite many advantages of the models, some limitations remain. First, the MACEs in our study only included all causes of death and HF readmission that were clear during our follow-up process. This led to incomplete analysis results. Second, the data of our study were mainly from the Shanxi Province of China, which limits generalizability and requires further validation in other populations. Finally, the clinical data was not included in the models of this study. In the following studies, we will establish a prognosis model using the data of clinical indicators and CHF-PRO in our further studies.

## Conclusion

Using variables of CHF-PRO and general information that could be obtained outside hospitals, we established prognosis models with good performance in patients with CHF via XGBoost. The self-made web-based risk calculator based on the models could serve as a convenient tool to predict the prognosis for out-of-hospital patients without some clinical indicators.

## Electronic supplementary material

Below is the link to the electronic supplementary material.

Supplementary Material 1

## Data Availability

Please contact the corresponding author for the study data, which will be granted upon reasonable request.

## Abbreviations

AUC: Area under curve
BMI: Body mass index
CHF: Chronic heart failure
CI: Confidence interval
LightGBM: Light gradient boosting machine
LR: Logistic regression
MACEs: Major adverse cardiovascular events
ML: Machine learning
MLP: Multilayer perceptron
NB: Naive bayes
NYHA: New York Heart Association
PHY: Physical domain
PROs: Patient-reported outcomes
PSY: Psychological domain
RFE: Recursive feature elimination
RF: Random forest
SD: Standard deviations
SHAP: Shapley additive explanations
SMOTE: Synthetic minority over-sampling technique algorithm
SOC: Social domain
XGBoost: Extreme gradient boosting
TRE: Therapeutic domain
