# Supplementary material for: Machine learning prognosis model based on patient-reported outcomes for chronic heart failure patients after discharge
Source: Health Qual Life Outcomes. 2023 Mar 29;21:31. doi: 10.1186/s12955-023-02109-x (PMC10053412; doi:10.1186/s12955-023-02109-x)
Supplement: Supplementary file 1 — Supplementary Material 1 [file 12955_2023_2109_MOESM1_ESM.pdf]

Table S1 TRIPOD Statement—Checklist of items

Section/Topic	Item	Checklist Item	Page
**Title and abstract**			
Title	1	Identify the study as developing and/or validating a multivariable prediction model, the target population, and the outcome to be predicted.	Title
Abstract	2	Provide a summary of objectives, study design, setting, participants, sample size, predictors, outcome, statistical analysis, results, and conclusions.	Abstract
**Introduction**			
Background and objectives	3a	Explain the medical context (including whether diagnostic or prognostic) and rationale for developing or validating the multivariable prediction model, including references to existing models.	1
	3b	Specify the objectives, including whether the study describes the development or validation of the model or both.	2
**Methods**			
Source of data	4a	Describe the study design or source of data (e.g., randomized trial, cohort, or registry data), separately for the development and validation data sets, if applicable.	2
	4b	Specify the key study dates, including start of accrual; end of accrual; and, if applicable, end of follow-up.	2
Participants	5a	Specify key elements of the study setting (e.g., primary care, secondary care, general population) including number and location of centres.	2
	5b	Describe eligibility criteria for participants.	2

	5c	Give details of treatments received, if relevant.	N/A
Outcome	6a	Clearly define the outcome that is predicted by the prediction model, including how and when assessed.	2, 4
	6b	Report any actions to blind assessment of the outcome to be predicted.	N/A
Predictors	7a	Clearly define all predictors used in developing or validating the multivariable prediction model, including how and when they were measured.	2, 3
	7b	Report any actions to blind assessment of predictors for the outcome and other predictors.	N/A
Sample size	8	Explain how the study size was arrived at.	4, 5
Missing data	9	Describe how missing data were handled (e.g., complete-case analysis, single imputation, multiple imputation) with details of any imputation method.	4
Statistical analysis methods	10a	Describe how predictors were handled in the analyses.	4
	10b	Specify type of model, all model-building procedures (including any predictor selection), and method for internal validation.	4, 5, 6
	10d	Specify all measures used to assess model performance and, if relevant, to compare multiple models.	6
Risk groups	11	Provide details on how risk groups were created, if done.	N/A
**Results**			
Participants	13a	Describe the flow of participants through the study, including the number of participants with and without the outcome and, if applicable, a summary of the follow-up time. A diagram may be helpful.	Table 1
	13b	Describe the characteristics of the participants (basic demographics, clinical	Table 1,

		features, available predictors), including the number of participants with missing data for predictors and outcome.	Table S3-S5
Model development	14a	Specify the number of participants and outcome events in each analysis.	Table 1
	14b	If done, report the unadjusted association between each candidate predictor and outcome.	Table S3-S5
Model specification	15a	Present the full prediction model to allow predictions for individuals (i.e., all regression coefficients, and model intercept or baseline survival at a given time point).	8, 9
	15b	Explain how to the use the prediction model.	9
Model performance	16	Report performance measures (with CIs) for the prediction model.	Table 2
**Discussion**			
Limitations	18	Discuss any limitations of the study (such as nonrepresentative sample, few events per predictor, missing data).	11
Interpretation	19b	Give an overall interpretation of the results, considering objectives, limitations, and results from similar studies, and other relevant evidence.	10, 11
Implications	20	Discuss the potential clinical use of the model and implications for future research.	10, 11
**Other information**			
Supplementary information	21	Provide information about the availability of supplementary resources, such as study protocol, Web calculator, and data sets.	Supplementary
Funding	22	Give the source of funding and the role of the funders for the present study.	Title page

Table S2 Structure of the CHF-PROM

Domain	Subdomain	Item
**Physical domain**	Somatic symptoms	PHD1-,PHD2-,PHD3-,PHD4-,PHD5-,PHD6-,PHD7-,PHD8-
	Appetite symptoms	PHD9-,PHD10-,PHD11-,PHD12-
	Independence	PHD13,PHD14,PHD15,PHD16
**Psychological domain**	Anxiety	PSD1-,PSD2-,PSD3-,PSD4-,PSD5-,PSD6-,PSD7-,PSD8-
	Depression	PSD9-,PSD10-,PSD11-,PSD12-,PSD13-,PSD14-
	Fear	PSD15-,PSD16-,PSD17-
	Paranoia	PSD18-,PSD19-,PSD20-,PSD21-
**Social domain**	Social support	SOC1,SOC2,SOC3,SOC4,SOC5
	Support utilization	SOC6,SOC7,SOC8
**Therapeutic domain**	Compliance	TRE1,TRE2
	Satisfaction	TRE3,TRE4,TRE5,TRE6,TRE7,TRE8,TRE9,TRE10
	Side effects	TRE11,TRE12-

CHF-PRO, chronic heart failure - patient reported outcome; PHY, physical domain; PSY, psychological domain; SOC, social domain; TRE; therapeutic domain.

Table S3 optimized hyperparameters of Machine Learning Methods

Parameter	Meaning	Distribution and search range	Final model configuration		
			**Death**	**Readission**	**MACEs**
**RF (RandomForestClassifier)**					
n_estimators	The number of trees in the forest	[100, 1000]	200	700	500
max_depth	Maximum tree depth for base learners	[3, 25]	17	13	15
min_samples_split	The minimum number of samples required to split an internal node	[2, 25)	6	2	2
min_samples_leaf	The minimum number of samples required to be at a leaf node	[1, 25)	10	10	10
min_weight_fraction_leaf	The minimum weighted fraction of the sum total of weights required to be at a leaf node	(0.1, 0.5)	0.4	0.15	0.3
max_features	The number of features to consider when looking for the best split	auto、sqrt、log2、None	14	8	12
**XGBoost (XGBClassifier package)**					
learning_rate	Boosting learning rate	[0.01, 1]	0.2	0.01	0.07
max_depth	Maximum tree depth for base learners	[3, 25]	15	10	5
min_child_weight	Minimum sum of instance weight(hessian) needed in a child	(0,+00）	0.6	1	0.5
n_estimators	Number of trees to fit	[100, 1000]	100	100	400
gamma	Minimum loss reduction required to make a further partition on a leaf node of the tree	(0,+	1	2	5
alpha	L1 regularization term on weights	(0,+	0.4	0.1	0.7
lambda	L2 regularization term on weights	(0,+	1	1	1
subsample	Subsample ratio of the training instance	[0.5, 1.0]	0.5	0.4	0.7
colsample_bytree	Subsample ratio of columns when constructing each tree	[0.5, 1.0]	0.6	0.75	0.86
**LightGBM (LGBMClassifier package)**					

learning_rate	Boosting learning rate	[0.01, 1]	0.1	0.01	0.01
max_depth	Maximum tree depth for base learners	[3, 25]	12	15	13
min_child_weight	Minimum sum of instance weight(hessian) needed in a child	(0,+	14	1	20
n_estimators	The number of trees in the forest	[100, 1000]	800	100	700
reg_lambda	L2 regularization term on weights	(0,+	1	0.3	0.5
reg_alpha	L1 regularization term on weights	(0,+	200	100	100
colsample_bytree	Subsample ratio of columns when constructing each tree	[0.5, 1.0]	0.6	0.5	0.3
subsample	Subsample ratio of the training instance	[0.5, 1.0]	0.6	0.7	0.5
**Logistic (LogsticRegression package)**					
penalty	Used to specify the norm used in the penalization	'l1', 'l2', 'elasticnet' or 'none'	l1	l1	l1
C	Inverse of regularization strength	(0, 1]	0.05	0.02	0.08
max_iter	Maximum number of iterations taken for the solvers to converge	[100, 1000]	100	100	100
**NB (BernoulliNB package)**					
alpha	Additive (Laplace/Lidstone) smoothing parameter (0 for no smoothing)	(0,+	0.0001	0.0001	0.0001
binarize	Threshold for binarizing (mapping to booleans) of sample features	（1, 200）	40	60	60
**MLP (MLPClassifier package)**					
hidden_layer_sizes	The ith element represents the number of neurons in the ith hidden layer.	(1, 300)	3	35	25
activation	Activation function for the hidden layer	'identity', 'logistic', 'tanh', 'relu'	'relu'	'relu'	'relu'
solver	The solver for weight optimization	'lbfgs', 'sgd', 'adam'	'adam'	'adam'	'adam'
alpha	L2 penalty	[0.0001,1)	0.01	0.13	0.03

	(regularization term) parameter				
learning_rate_init	It controls the step-size in updating the weights	[0.01, 1]	0.03	0.02	0.01
max_iter	Maximum number of iterations	[100,1000]	600	100	100
beta_1	Exponential decay rate for estimates of first moment vector in adam	[0.001, 1)	0.42	0.65	0.61
beta_2	Value for numerical stability in adam	[0.001, 1)	0.07	0.05	0.44

Table S4 Baseline Characteristics of Patients With CHF Included in the Study

	Training set	Testing set	*P*
**Death**	44 (6.50%)	21 (7.95%)	0.429
**HF Readmission**	198 (29.25%)	70 (26.52%)	0.404
**MACEs**	237 (35.01%)	89 (33.71%)	0.708
**Age**	68.87 (58.7, 78.18)	67.67 (58.29, 77.25)	0.516
**Female**	293 (43.28%)	106 (40.15%)	0.383
**NYHA**			≤0.001
II	184 (27.18%)	2 (0.76%)	
III	284 (41.95%)	102 (38.64%)	
IV	209 (30.87%)	160 (60.61%)	
**Heart rate (beats per minute)**	76 (68, 88)	78 (65, 90)	0.999
**Systolic blood pressure (mmHg)**	125 (114, 139)	120 (108, 138)	0.002
**Diastolic blood pressure (mmHg)**	76 (68, 84)	72 (66, 82)	0.094
**Body Mass Index (kg/m^2)**	23.5 (21.30, 26.22)	23.44 (20.31, 27.14)	0.798
**Marital status**			0.529
Married	12 (1.77%)	5 (1.89%)	
Single	558 (82.42%)	213 (80.68%)	
Divorced	7 (1.03%)	6 (2.27%)	
Widowed	100 (14.77%)	40 (15.15%)	
**Education**			0.036
Illiteracy	59 (8.71%)	22 (8.33%)	

Low level	387 (57.16%)	174 (65.91%)	
Secondary school and high level	231 (34.12%)	68 (25.76%)	
**Occupation**			0.073
Manual workers	438 (64.70%)	187 (70.83%)	
Nonmanual workers	239 (35.30%)	77 (29.17%)	
**Income**			0.061
Low	363 (53.62%)	162 (61.36%)	
Medium	301 (44.46%)	100 (37.88%)	
High	13 (1.92%)	2 (0.76%)	
**Health care**			0.585
City health insurance	432 (63.81%)	168 (63.64%)	
Rural health insurance	218 (32.20%)	89 (33.71%)	
Self-paying	27 (3.99%)	7 (2.65%)	
**Family history**	181 (26.74%)	60 (22.73%)	0.206
**Smoking history**			0.106
Never	428 (63.22%)	149 (56.44%)	
Quitting smoking	164 (24.22%)	81 (30.68%)	
Smoking	85 (12.56%)	34 (12.88%)	
**Drinking**	47 (6.94%)	15 (5.68%)	≤0.001
**Complications**			
Diabetes	197 (29.10%)	65 (24.62%)	0.169

Atrial fibrillation	233 (34.42%)	120 (45.45%)	0.002
Stroke	98 (14.48%)	69 (26.14%)	≤0.001
COPD	94 (13.88%)	20 (7.58%)	0.008
Cancers	30 (4.43%)	1 (0.38%)	0.002
Coronary heart disease	363 (53.62%)	124 (46.97%)	0.067
Hypertension	396 (58.49%)	121 (45.83%)	≤0.001
Chronic renal insufficiency	135 (19.94%)	49 (18.56%)	0.631
Valvular heart disease	127 (18.76%)	71 (26.89%)	0.006
**CHF-PRO**			
Physical domain	41 (33, 49)	40 (32, 49)	0.191
Psychological domain	69 (56, 77)	70 (58, 78)	0.404
Social domain	18 (13, 22)	17 (13, 21)	0.135
Therapeutic domain	34 (31, 39)	34 (31, 38)	0.432

CHF-PRO, chronic heart failure - patient reported outcome; COPD, chronic obstructive pulmonary disease; NYHA, New York Heart Association functional class; MACEs, major adverse cardiovascular events.

Table S5 Baseline Characteristics of Death and Survival Patients

	Survival (n=876)	Death (n=65)	*P*
**Age**	67.83 (58.24, 77.66)	73.84 (64.83, 80.34)	0.009
**Female**	507 (57.88%)	35 (53.85%)	0.526
**NYHA**			＜0.001
II	176 (20.09%)	10 (15.38%)	
III	372 (42.47%)	14 (21.54%)	
IV	328 (37.44%)	41 (63.08%)	
**Heart rate (beats per minute)**	76 (67, 89)	77 (68, 92)	0.318
**Systolic blood pressure (mmHg)**	125 (112, 139)	118 (106, 132)	0.005
**Diastolic blood pressure (mmHg)**	76 (68, 84)	72 (62, 81)	0.092
**Body Mass Index (kg/m^2)**	23.66 (21.22, 26.67)	21.85 (19.68, 24.68)	0.002
**Marital state**			0.258
Married	15 (1.71%)	2 (3.08%)	
Single	722 (82.42%)	49 (75.38%)	
Divorced	13 (1.48%)	0	
Widowed	126 (14.38%)	14 (21.54%)	
**Education**			0.963
Illiteracy	76 (8.68%)	5 (7.69%)	
Low level	522 (59.59%)	39 (60.00%)	
Secondary school and high level	278 (31.74%)	21 (32.31%)	
**Occupation**			0.297

Manual workers	578 (65.98%)	47 (72.31%)	
Nonmanual workers	298 (34.02%)	18 (27.69%)	
**Income**			0.997
Low	489 (55.82%)	36 (55.38%)	
Medium	373 (42.58%)	28 (43.08%)	
High	14 (1.60%)	1 (1.54%)	
**Health care**			0.955
City health insurance	559 (63.81%)	41 (63.08%)	
Rural health insurance	285 (32.53%)	22 (33.85%)	
Self-paying	32 (3.65%)	2 (3.08%)	
**Family history**	231 (26.37%)	10 (15.38%)	0.050
**Smoking history**			0.531
Never	538 (61.42%)	39 (60.00%)	
Quitting smoking	230 (26.26%)	15 (23.08%)	
Smoking	108 (12.33%)	11 (16.92%)	
**Drinking**	59 (6.74%)	3 (4.62%)	0.129
**Complications**			
Coronary heart disease	450 (51.37%)	37 (56.92%)	0.387
Hypertension	488 (55.71%)	29 (44.62%)	0.083
Chronic renal insufficiency	164 (18.72%)	20 (30.77%)	0.018
Diabetes	244 (27.85%)	18 (27.69%)	0.978
Atrial fibrillation	324 (36.99%)	29 (44.62%)	0.220

Stroke	157 (17.92%)	10 (15.38%)	0.605
COPD	106 (12.10%)	8 (12.31%)	0.961
Cancers	26 (2.97%)	5 (7.69%)	0.040
Valvular heart disease	181 (20.66%)	17 (26.15%)	0.295
**CHF-PRO**			
Physical domain	42 (33, 50)	35 (28, 40)	＜0.001
Psychological domain	69 (56, 77)	67 (58, 78)	0.911
Social domain	17 (13, 21)	18 (14, 22)	0.270
Therapeutic domain	34 (31, 39)	34 (31, 41)	0.958

CHF-PRO, chronic heart failure - patient reported outcome; COPD, chronic obstructive pulmonary disease; NYHA, New York Heart Association functional class.

Table S6 Baseline Characteristics of Patients With or Without Rehospitalization

	**Without Rehospitalization (n=673)**	**Rehospitalization (n=268)**	***P***
**Age**	67.59 (56.64, 77.84)	70.09 (62.05, 78.05)	0.040
**Female**	267 (39.67%)	132 (49.25%)	0.007
**NYHA**			0.579
II	136 (20.21%)	50 (18.66%)	
III	269 (39.97%)	117 (43.66%)	
IV	268 (39.82%)	101 (37.69%)	
**Heart rate (beats per minute)**	77 (67, 90)	75 (66, 87)	0.185
**Systolic blood pressure (mmHg)**	124 (111, 138)	125 (110, 139)	0.919
**Diastolic blood pressure (mmHg)**	75 (67, 83)	76 (67, 83)	0.811
**Body Mass Index (kg/m^2)**	23.44 (20.90, 26.56)	23.73 (21.45, 26.57)	0.420
**Marital status**			0.829
Married	12 (1.78%)	5 (1.87%)	
Single	555 (82.47%)	216 (80.60%)	
Divorced	8 (1.19%)	5 (1.87%)	
Widowed	98 (14.56%)	42 (15.67%)	
**Education**			0.559
Illiteracy	55 (8.17%)	26 (9.70%)	
Low level	408 (60.62%)	153 (57.09%)	

Secondary school and high level	210 (31.20%)	89 (33.21%)	
**Occupation**			0.540
Manual workers	451 (67.01%)	174 (64.93%)	
Nonmanual workers	222 (32.99%)	94 (35.07%)	
**Income**			0.329
Low	384 (57.06%)	141 (52.61%)	
Medium	280 (41.60%)	121 (45.15%)	
High	9 (1.34%)	6 (2.24%)	
**Health care**			0.273
City health insurance	419 (62.26%)	181 (67.54%)	
Rural health insurance	230 (34.18%)	77 (28.73%)	
Self-paying	24 (3.57%)	10 (3.73%)	
**Family history**	170 (25.26%)	71 (26.49%)	0.696
**Smoking history**			0.004
Never	391 (58.10%)	186 (69.40%)	
Quitting smoking	187 (27.79%)	58 (21.64%)	
Smoking	95 (14.12%)	24 (8.96%)	
**Drinking**	47 (6.98%)	15 (5.60%)	0.007
**Complications**			
Coronary heart disease	350 (52.01%)	137 (51.12%)	0.806
Hypertension	368 (54.68%)	149 (55.60%)	0.799
Chronic renal insufficiency	124 (18.42%)	60 (22.39%)	0.167

Diabetes	183 (27.19%)	79 (29.48%)	0.480
Atrial fibrillation	237 (35.22%)	116 (43.28%)	0.021
Stroke	112 (16.64%)	55 (20.52%)	0.160
COPD	84 (12.48%)	30 (11.19%)	0.585
Cancers	21 (3.12%)	10 (3.73%)	0.636
Valvular heart disease	133 (19.76%)	65 (24.25%)	0.127
**CHF-PRO**			
Physical domain	42 (33, 50)	39 (31, 46)	＜0.001
Psychological domain	71 (58, 78)	65 (52, 74)	＜0.001
Social domain	17 (13, 21)	17 (13, 21)	0.283
Therapeutic domain	34 (31, 39)	34 (31, 39)	0.954

CHF-PRO, chronic heart failure - patient reported outcome; COPD, chronic obstructive pulmonary disease; NYHA, New York Heart Association functional class.

Table S7 Baseline Characteristics of Patients With or Without MACEs

	Without-MACEs (n=615)	MACEs (n=326)	*P*
**Age**	67.19 (56.38, 77.56)	71.42 (62.32, 78.96)	0.002
**Female**	242 (39.35%)	157 (48.16%)	0.009
**NYHA**			0.354
II	128 (20.81%)	58 (17.79%)	
III	255 (41.46%)	131 (40.18%)	
IV	232 (37.72%)	137 (42.02%)	
**Heart rate (beats per minute)**	77 (67, 90)	75 (67, 88)	0.552
**Systolic blood pressure (mmHg)**	125 (112, 139)	122 (110, 136)	0.173
**Diastolic blood pressure (mmHg)**	76 (68, 84)	75 (66, 83)	0.28
**Body Mass Index (kg/m^2)**	23.5 (20.98, 26.84)	23.44 (21.09, 26.12)	0.466
**Marital status**			0.649
Married	10 (1.63%)	7 (2.15%)	
Single	511 (83.09%)	260 (79.75%)	
Divorced	8 (1.30%)	5 (1.53%)	
Widowed	86 (13.98%)	54 (16.56%)	
**Education**			0.588
Illiteracy	51 (8.29%)	30 (9.20%)	
Low level	374 (60.81%)	187 (57.36%)	
Secondary school and high level	190 (30.89%)	109 (33.44%)	

**Occupation**			0.939
Manual workers	409 (66.50%)	216 (66.26%)	
Nonmanual workers	206 (33.50%)	110 (33.74%)	
**Income**			0.440
Low	350 (56.91%)	175 (53.68%)	
Medium	257 (41.79%)	144 (44.17%)	
High	8 (1.30%)	7 (2.15%)	
**Health care**			0.316
City health insurance	382 (62.11%)	218 (66.87%)	
Rural health insurance	211 (34.31%)	96 (29.45%)	
Self-paying	22 (3.58%)	12 (3.68%)	
**Family history**	163 (26.50%)	78 (23.93%)	0.389
**Smoking history**			0.018
Never	357 (58.05%)	220 (67.48%)	
Quitting smoking	173 (28.13%)	72 (22.09%)	
Smoking	85 (13.82%)	34 (10.43%)	
**Drinking**	44 (7.15%)	18 (5.52%)	＜0.001
**Complications**			
Coronary heart disease	316 (51.38%)	171 (52.45%)	0.754
Hypertension	343 (55.77%)	174 (53.37%)	0.482
Chronic renal insufficiency	106 (17.24%)	78 (23.93%)	0.014
Diabetes	166 (26.99%)	96 (29.45%)	0.424

Atrial fibrillation	211 (34.31%)	142 (43.56%)	0.005
Stroke	103 (16.75%)	64 (19.63%)	0.271
COPD	77 (12.52%)	37 (11.35%)	0.601
Cancers	16 (2.60%)	15 (4.60%)	0.102
Valvular heart disease	120 (19.51%)	78 (23.93%)	0.114
**CHF-PRO**			
Physical domain	43 (34, 51)	38 (31, 45)	＜0.001
Psychological domain	71 (58, 78)	66 (53, 75)	＜0.001
Social domain	17 (13, 21)	17 (13, 21)	0.704
Therapeutic domain	34 (31, 39)	34 (31, 39)	0.834

CHF-PRO, chronic heart failure - patient reported outcome; COPD, chronic obstructive pulmonary disease; NYHA, New York Heart Association functional class; MACEs, major adverse cardiovascular events.

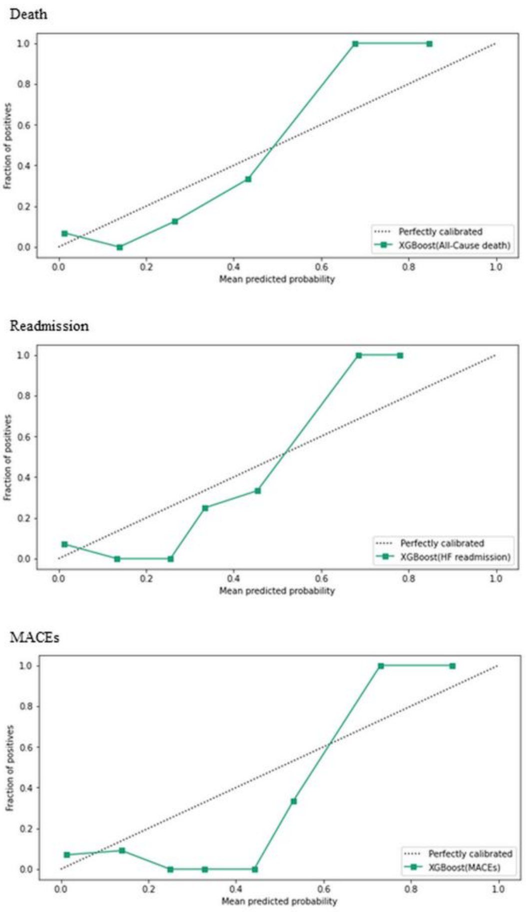

Figure S1. The calibration curves for predicting the outcomes of death (A), readmission (B) and MACEs (C). The x-axis represents the overall predicted probability of the outcomes and the y-axis represents the actual probability. Model calibration is indicated by the degree of fitting of curve and the diagonal.
